# Identifying Diabetic Kidney Disease in Type 2 Diabetes Patients Using Explainable Machine Learning: A Case‐Control Study

**DOI:** 10.1155/jdr/7196309

**Published:** 2026-06-07

**Authors:** Tongtong Qiu, Yi Bai, Hai Zhao, Mengyao Huang, Ping Zhang, Yu Zhang, Ruoyu Wang, Liqin An

**Affiliations:** ^1^ Department of Clinical Laboratory, Shaanxi Provincial People’s Hospital, Xi’an, Shaanxi, China, spph-sx.com; ^2^ Department of Information Center, Shaanxi Provincial People’s Hospital, Xi’an, Shannxi, China, spph-sx.com

**Keywords:** diabetic kidney disease, LIME, machine learning model, random forest, SHAP

## Abstract

**Objective:**

This research focused on establishing and testing a machine learning–driven predictive tool aimed at assisting in the identification of diabetic kidney disease (DKD).

**Methods:**

The prediction models were developed and internally temporally validated using single institution data. A total of 1463 patients from Shaanxi Provincial People’s Hospital between March 2023 and September 2024 were incorporated in our study. Least absolute shrinkage and selection operator regression with 10‐fold cross‐validation was used to select the optimal features. We compared extreme gradient boosting, random forest (RF), support vector machine, and logistic regression across a range of metrics: area under the receiver operating characteristic curve (AUC‐ROC), area under the precision‐recall curve (AUC‐PR), accuracy, precision, recall, kappa values, and F1‐score. For each algorithm, a simplified model was developed using only routinely available clinical variables and was trained and evaluated on the same datasets as the full model. Decision curve analysis and calibration curve served to evaluate the clinical utility of the optimal models. Analysis and interpretation of feature importance were performed via SHapley Additive exPlanations and Local Interpretable Model‐agnostic Explanations.

**Results:**

When screening for DKD in Type 2 diabetes, the full RF model achieved superior performance (AUC‐ROC = 0.906, AUC‐PR = 0.902, accuracy = 0.830, F1 = 0.847, precision = 0.794, recall = 0.907, and kappa = 0.657) and significantly outperformed the simplified RF model. It also exhibited a favorable clinical net benefit and well‐calibrated performance. The most influential predictors identified in the full RF model were urine *α*1‐microglobulin, hypertension, 24‐h urinary total protein, duration of Type 2 diabetes mellitus, systolic blood pressure, serum retinol‐binding protein, complement C1q, and 25‐hydroxyvitamin D.

**Conclusion:**

A RF prediction model was developed to facilitate early screening of DKD, highlighting the significant roles of specific clinical and laboratory factors in disease prediction.

## 1. Introduction

A substantial proportion of individuals with diabetes mellitus, ranging from 25% to 40%, are susceptible to developing diabetic kidney disease (DKD). This condition is defined by detrimental pathological modifications in both the architecture and physiological performance of the kidneys [1, 2]. The predominance of DKD as an underlying driver of CKD is well established, and its association with elevated rates of adverse events and death in diabetic cohorts is undeniable [3]. Evidence indicates that prompt intervention at the earliest discernible phase of DKD may forestall irreversible loss of kidney function and correspondingly improve survival metrics [4]. However, the onset and progression of DKD are governed by a multitude of intricate pathophysiological mechanisms, encompassing metabolic dysregulation, genetic factors, inflammation, and oxidative stress, which lead to challenges in clinical diagnosis and treatment [5].

Albuminuria and estimated glomerular filtration rate (eGFR) serve as the cornerstone prognostic markers for DKD advancement in routine care [6]. However, a substantial proportion of Type 2 diabetes (T2D) patients with impaired kidney function present without albuminuria, indicating a dissociation between declining kidney function and urinary albumin leakage [7]. Consequently, albuminuria proves an insufficient metric for forecasting DKD initiation and progression. Limitations of utilizing eGFR to predict disease progression include the relatively high margin of error associated with eGFR calculation formulas [8]. Furthermore, as creatinine is derived primarily from muscle tissue, creatinine‐based eGFR equations may yield inaccurate assessments of actual renal function in individuals with reduced muscle mass. Over the past decade, extensive research has been dedicated to identifying novel biomarkers for DKD [9]. However, given the heterogeneous nature and complex pathophysiology of DKD, it is unlikely that any single biomarker can sufficiently predict disease prognosis. Instead, a multimarker approach is likely necessary to accurately anticipate disease progression. Therefore, developing and validating innovative strategies for early detection and management of DKD is indispensable for improving patient outcomes.

In recent years, machine learning (ML) has been increasingly utilized in many biomedical applications as a promising tool to aid in decision‐making across various diseases. ML has been shown in multiple investigations to confer improvements upon diagnostic systems, specifically by strengthening the reliability, elevating predictive power, and sharpening accuracy [10]. For instance, Kaya et al. developed an XGBoost classifier utilizing maternal sociodemographic and obstetric history data to effectively predict gestational diabetes mellitus [11]. Similarly, ML‐based analysis of laboratory data has emerged as a valuable approach for identifying individuals at risk of developing T2D [12]. Multiple investigations have further deployed ML algorithms to forecast chronic kidney disease [13, 14]. Nonetheless, these efforts predominantly utilize routinely collected variables and seldom incorporate emerging biomarkers. Moreover, the incremental benefit conferred by complex ML architectures over parsimonious models restricted to basic clinical inputs remains inadequately characterized, and systematic comparisons between comprehensive and reduced models are scarce, leaving unresolved whether the inclusion of less accessible biomarkers yields clinically relevant prognostic gains. To bridge this gap, we aimed to develop and validate a robust ML framework that integrates multidimensional patient profiles for the early detection of DKD. Specifically, this study is aimed at (1) developing and contrasting both full and simplified ML models within identical algorithmic frameworks and (2) identifying comparatively novel biomarkers that serve as robust predictors.

## 2. Materials and Methods

### 2.1. Data Sources and Study Population

A case‐control study was undertaken involving Chinese patients diagnosed with T2D who were recruited from Shaanxi Provincial People’s Hospital. Ethical clearance was obtained from the Shaanxi Provincial People’s Hospital Ethics Review Board (Approval No. SPPH‐LLBG‐34‐4.0‐2025R078), and all procedures adhered to the principles stipulated in the Declaration of Helsinki. We obtained data through the Electronic Health Medical Record Management System (EHRMS) of Shaanxi Provincial People’s Hospital. The training dataset, consisting of 1046 participants, was obtained from March 2023 to March 2024. The test dataset, comprising 417 participants, was collected from April 2024 to September 2024 at the Shaanxi Provincial People’s Hospital. This was a model development study with internal validation using a temporal train‐test split within a single center. No external validation was conducted. The inclusion criteria comprised participants aged > 18 years who were diagnosed with either T2D complicated by DKD or T2D without DKD. The diagnostic classification of the recruited participants was determined in accordance with the criteria set forth in the Standards of Medical Care in Diabetes‐2022 [15]. A clinical diagnosis of DKD was established based on persistent albuminuria (urinary albumin‐to‐creatinine ratio [UACR] ≥ 30 mg/g) and/or reduced eGFR (< 60 mL/min/1.73 m^2^) persisting for more than 3 months, after excluding other causes of kidney damage. Alternative or concurrent renal pathologies should be suspected when active urinary sediment (evidenced by red blood cells, white blood cells, or casts), rapidly worsening albuminuria or proteinuria, nephrotic‐range protein loss, or a sharp eGFR decrement is observed [16]. Exclusion from the study applied to patients with Type 1 diabetes, gestational diabetes, other types of diabetes, acute diabetic complications, renal diseases other than DKD, malignancy, or insufficient available clinical data.

### 2.2. Data Collection

Demographic characteristics, anthropometric measurements, laboratory parameters, and relevant comorbidities were retrieved from the subjects’ medical records via an electronic healthcare information system. A total of 56 variables were taken into account in the study, namely, age, gender, height, weight, duration of T2D, systolic blood pressure (SBP), diastolic blood pressure (DBP), body temperature, pulse rate (PR), respiratory rate (RR), body mass index (BMI), hypertension, diabetic retinopathy (DR), diabetic peripheral vascular disease (DPVD), diabetic peripheral neuropathy (DPN), dyslipidemia, serum 25‐hydroxyvitamin D (25(OH)D), complement C1q, cystatin C (Cys‐C), neutrophil gelatinase‐associated lipocalin (NGAL), retinol‐binding protein (RBP), serum *β*2‐microglobulin (*β*2‐MG), serum albumin, high‐sensitivity cardiac troponin T (hs‐cTnT), alanine aminotransferase (ALT), aspartate aminotransferase (AST), thyroid‐stimulating hormone (TSH), total bilirubin (TBIL), high‐density lipoprotein cholesterol (HDL‐C), low‐density lipoprotein cholesterol (LDL‐C), total cholesterol (TC), triglyceride (TG), hemoglobin A1c (HbAc1), fasting blood glucose (FBG), phosphate (P), sodium (Na), potassium (K), chlorine (Cl), calcium (Ca), blood urea nitrogen (BUN), uric acid (UA), creatinine, 24‐h urine total protein (UTP), urine a1‐microglobulin (a1‐MG), urine *β*2‐MG, immunoglobulin G (IgG), urine albumin, transferrin, UACR, plasma fibrinogen (FIB), D‐dimer, fibrinogen degradation products (FDPs), hemoglobin, neutrophil percentage, platelet (PLT) count, and eGFR. BMI was calculated as weight (kilogram) divided by height squared (square meter), and eGFR was estimated via the 2021 CKD‐EPI creatinine equation [17].

### 2.3. Data Cleaning and Feature Selection

**Figure 1:**
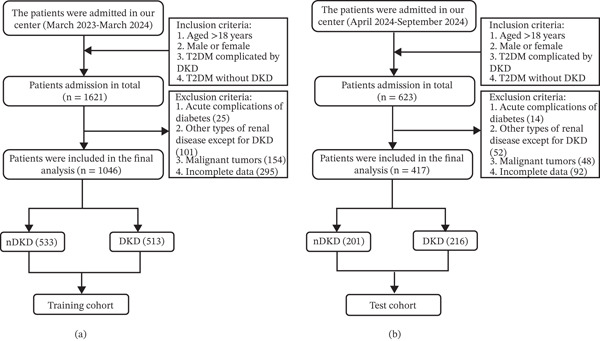
Schematic diagram of data preprocessing.

The prediction model treats the onset of DKD as a dichotomous variable, coded as 1 to indicate the presence of disease and 0 to indicate its absence. Features with > 50% missing values and observations with any missing data were excluded from analysis (preprocessing steps summarized in Figure 1). To examine the influence of missing data handling, a sensitivity analysis with median imputation was undertaken, and area under the receiver operating characteristic curve (AUC‐ROC) values were contrasted between the complete‐case and imputed datasets.

Features were categorized as either continuous or binary in the present study. Continuous predictors were standardized using the “scale” function in R, with the mean and standard deviation parameters derived exclusively from the training data and subsequently applied to the test set. Feature selection was conducted solely on the training set via least absolute shrinkage and selection operator (LASSO) regression. This approach employs L1‐norm regularization to penalize coefficient magnitudes, progressively shrinking noninformative predictors to zero and thereby retaining the subset most predictive of DKD onset. The optimal regularization hyperparameter (*λ*) was determined through 10‐fold cross‐validation, selecting the value associated with the minimum mean cross‐validated error.

### 2.4. Construction and Evaluation of ML Models

Using the key features selected as described, four DKD prediction models were constructed using the following classification algorithms: extreme gradient boosting (XGBoost), random forest (RF), support vector machine (SVM) based on radial basis function, and logistic regression. These algorithms were implemented via the R packages “xgboost,” “randomForest,” “e1071,” and “glmnet,” respectively. Hyperparameter optimization for each model was conducted exclusively on the training data through a grid search procedure coupled with 10‐fold cross‐validation. All models were subsequently trained with their respective optimal hyperparameter configurations and assessed for generalizability and clinical utility on the independent test dataset.

To assess whether our proposed models offer added value over current clinical practice, we developed four simple ML models using only variables that are routinely available in outpatient or inpatient settings without requiring specialized assays or computational processing. These variables included age, gender, duration of T2D, hypertension, eGFR, and UACR. The simplified models were trained on the same training set and evaluated on the same test set as the four proposed models. The generalization ability of the eight models was assessed by the following performance metrics: AUC‐ROC, area under the precision‐recall curve (AUC‐PR), precision, accuracy, recall, kappa values, and F1‐score. Precision = TP/(TP + FP), recall = TP/(TP + FN), and F1 − score = 2TP/(2TP + FP + FN), where true positive (TP) is the number of correctly classified patients with DKD, false positive (FP) is the number of incorrectly classified patients without DKD, and false negative (FN) is the number of incorrectly classified patients with DKD.

The clinical utility of the models was evaluated through the application of the decision curve analysis (DCA) and calibration curve. DCA was performed using the “dcurves” R package. Net benefit was calculated for the optimal full model and the optimal simple model across a range of clinically reasonable threshold probabilities and compared with two extreme strategies: “treat all” and “treat none.” Decision curves were plotted with threshold probability on the *x*‐axis and net benefit on the *y*‐axis. Calibration curves were generated by grouping test set predictions into eight equally spaced bins, plotting the observed event rate against the mean predicted probability, with a 45° diagonal line representing perfect calibration. To gain insight into how input features influence model outputs, SHapley Additive exPlanations (SHAP) values and the Local Interpretable Model‐agnostic Explanations (LIME) method were employed for interpretability analysis.

### 2.5. Statistical Analysis

**Figure 2:**
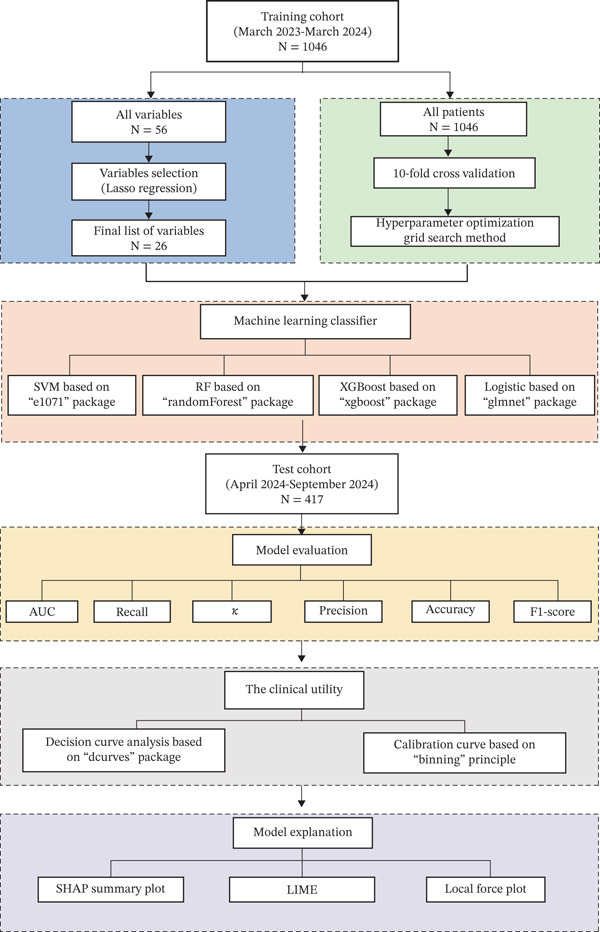
DKD statistical analysis workflow diagram. The process encompasses four machine learning classifiers, hyperparameter tuning of classifiers using grid search, and final model evaluation and explanation.

Statistical computations were carried out using R (Version 4.5.2) and GraphPad Prism 8. Continuous variables were summarized as median values accompanied by the interquartile range (25th–75th percentiles). Between‐group comparisons of clinical parameters were conducted via the Mann–Whitney *U* test. Associations were assessed using Spearman’s rank correlation. Categorical frequency differences were evaluated with the chi‐squared test. All tests were two‐tailed, and statistical significance was defined as a *p* value below 0.05. The overall analytic workflow is illustrated in Figure 2.

## 3. Results

### 3.1. Feature Selection

**Figure 3:**
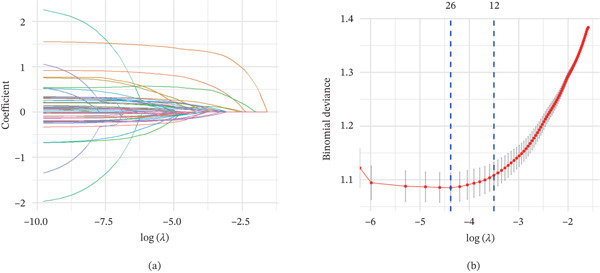
(a) Each feature’s LASSO regression coefficient; (b) the value of *λ* with the smallest mean error was determined through 10‐fold cross‐validation. (a) Each line corresponds to the coefficient estimate of a feature, with each estimated parameter exhibiting a monotonic decrease as *λ* increases, ultimately converging to zero. (b) The relationship between the binomial deviance and log (*λ*) was plotted. Vertical dashed lines indicate the optimal value selected by the minimum criterion and the 1SE principle. The *λ* value of 0.013 was determined through 10‐fold cross‐validation, with the optimal feature count identified as 26.

**Figure 4:**
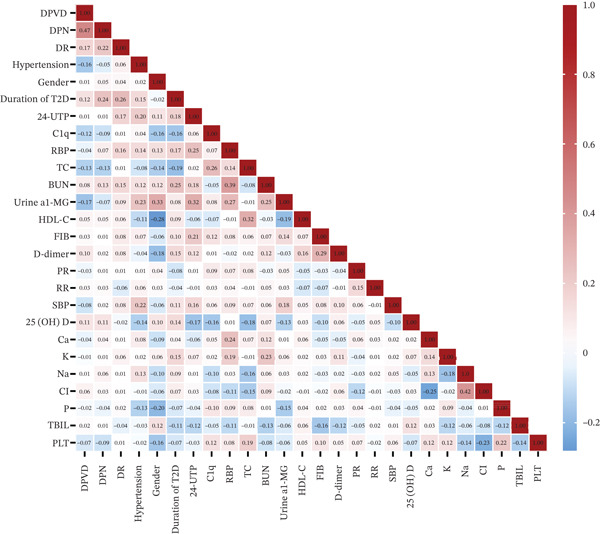
Heatmap of the correlations among key features. DPN, diabetic peripheral neuropathy; DPVD, diabetic peripheral vascular disease; DR, diabetic retinopathy; RR, respiratory rate; PR, pulse rate; SBP, systolic blood pressure; 25(OH)D, 25‐hydroxyvitamin D; HDL‐C, high‐density lipoprotein cholesterol; RBP, retinol‐binding protein; TC, total cholesterol; BUN, blood urea nitrogen; 24‐h UTP, 24‐h urine total protein; a1‐MG, a1‐microglobulin; TBIL, total bilirubin; P, phosphate; Na, sodium; K, potassium; Cl, chlorine; Ca, calcium; FIB, fibrinogen; PLT, platelet.

In total, 1046 patients with 56 features were included in the training set and assigned to one of the two groups (DKD or nDKD) based on the clinical diagnosis. Using the “glmnet” package implementation in R language, the most predictive features were screened from 56 features to reduce the dimensionality (Figure 3). After LASSO regression analysis, 26 key features were selected, including gender, DPN, DPVD, DR, hypertension, PR, RR, SBP, serum 25(OH)D, serum C1q, HDL‐C, RBP, serum TC, BUN, serum TBIL, serum P, Na, K, Cl, Ca, 24‐h UTP, urine a1‐MG, duration of T2D, plasma FIB, plasma D‐dimer, and PLT count. Additionally, the Spearman correlation coefficients among these 26 variables are relatively low, indicating the absence of significant multicollinearity (Figure 4).

### 3.2. Characteristics of the Study Population

The final size of the training dataset was obtained as 1046 samples × 26 features, while the test dataset comprised 417 samples × 26 features. This sample size was sufficient to meet the requisite statistical criteria and uphold the robustness of the study conclusions [18]. The clinical characteristics of the participants stratified by DKD status are presented in Tables 1 and 2. Nearly all but a few variables exhibited statistical differences between the nDKD and DKD groups, indicating that the features may hold significant potential for identifying patients with DKD among those with diabetes.

**Table 1:** Baseline characteristics of the training dataset.

	nDKD (533)	DKD (513)	*p* value
Male gender, *n* (%)	321 (60.2%)	359 (70.0%)	< 0.05
DPN, *n* (%)	265(49.7%)	280 (54.6%)	0.13
DPVD, *n* (%)	316 (59.3%)	252 (49.1%)	< 0.05
DR, *n* (%)	47 (8.8%)	121 (23.6%)	< 0.05
Hypertension, *n* (%)	241 (45.2%)	433 (84.4%)	< 0.05
25(OH)D (ng/mL)	15.87 (11.46–21.96)	13.60 (8.83–19.23)	< 0.05
C1q (mg/L)	146.4 (124.5–174.5)	154.9 (134.4–180.5)	< 0.05
D‐dimer (mg/L)	0.64 (0.55–0.79)	0.63 (0.53–0.77)	0.12
HDL‐C (mmol/L)	1.13 (0.97–1.32)	1.07 (0.91–1.27)	< 0.05
RBP (mg/L)	41.50 (34.40–48.41)	47.13 (37.90–58.19)	< 0.05
TC (mmol/L)	4.20 (3.39–5.00)	4.00 (3.17–4.96)	0.10
BUN (mmol/L)	5.63 (4.68–6.67)	6.20 (5.13–7.58)	< 0.05
24‐h UTP (mg/24 h)	126.9 (81.5–210.7)	220.4 (132.3–417.9)	< 0.05
RR (times/min)	20 (20–20)	20 (20–20)	< 0.05
Urine a1‐MG (*μ*g/mL)	5.73 (3.33–10.21)	11.85 (7.06–22.61)	< 0.05
TBIL (*μ*mol/L)	12.30 (9.80–15.77)	11.70 (8.71–15.23)	< 0.05
SBP (mmHg)	126 (116–138)	135 (120–147)	< 0.05
P (mmol/L)	1.17 (1.05–1.32)	1.14 (1.03–1.25)	< 0.05
Cl (mmol/L)	104 (103–107)	104 (102–106)	0.14
Duration of T2D (years)	9 (5–15)	13 (8–19)	< 0.05
FIB (g/L)	3.03 (2.63–3.62)	3.11 (2.69–3.66)	0.22
PR (times/min)	82 (75–90)	84 (76–95)	< 0.05
PLT (10^9^/L)	189 (158–232)	188 (155–228)	0.29
Ca (mmol/L)	2.26 (2.17–2.35)	2.30 (2.20–2.37)	< 0.05
Na (mmol/L)	141 (139–142)	141 (140–143)	< 0.05
K (mmol/L)	3.9 (3.7–4.1)	4.0 (3.7–4.2)	< 0.05

*Note:* Continuous variables are presented as median (25th–75th percentile), with *p* value calculated using the Mann–Whitney *U* test. Categorical variables are expressed as percentages, with *p* value determined by a chi‐square test.

Abbreviations: 24‐h UTP, 24‐h urine total protein; 25(OH)D, 25‐hydroxyvitamin D; a1‐MG, a1‐microglobulin; BUN, blood urea nitrogen; Ca, calcium; Cl, chlorine; DPN, diabetic peripheral neuropathy; DPVD, diabetic peripheral vascular disease; DR, diabetic retinopathy; FIB, fibrinogen; HDL‐C, high‐density lipoprotein cholesterol; K, potassium; Na, sodium; P, phosphate; PLT, platelets; PR, pulse rate; RBP, retinol‐binding protein; RR, respiratory rate; SBP, systolic blood pressure; TBIL, total bilirubin; TC, total cholesterol.

**Table 2:** Baseline characteristics of the test dataset.

	nDKD (201)	DKD (216)	*p* value
Male gender, *n* (%)	112 (55.7%)	154 (71.3%)	< 0.05
DPN, *n* (%)	36 (17.9%)	111 (51.4%)	< 0.05
DPAD, *n* (%)	34 (16.9%)	85 (39.4%)	< 0.05
DR, *n* (%)	13 (6.5%)	64 (29.6%)	< 0.05
Hypertension, *n* (%)	195 (97.0%)	212 (98.1%)	0.53
25(OH)D (ng/mL)	15.09 (9.16–22.28)	13.34 (8.24–20.15)	0.07
C1q (mg/L)	150.4 (127.1–173.8)	162.4 (138.8–191.4)	< 0.05
D‐dimer (mg/L)	0.60 (0.51–0.74)	0.59 (0.47–0.79)	0.36
HDL‐C (mmol/L)	1.17 (1.01–1.35)	1.11 (0.89–1.27)	< 0.05
RBP (mg/L)	38.70 (31.35–44.09)	49.47 (37.06–62.58)	< 0.05
TC (mmol/L)	4.12 (3.43–4.83)	3.94 (3.21–4.91)	0.52
BUN (mmol/L)	5.47 (4.69–6.48)	6.7 (5.43–8.23)	< 0.05
24‐h UTP (mg/24 h)	109.2 (76.0–174.0)	209.1 (108.0–446.7)	< 0.05
RR (times/min)	20 (20–20)	20 (19–20)	0.84
Urine a1‐MG (*μ*g/mL)	5.24 (2.81–9.94)	13.08 (7.68–28.52)	< 0.05
TBIL (*μ*mol/L)	12.60 (10.10–15.58)	11.25 (8.53–14.85)	< 0.05
SBP (mmHg)	126 (114–135)	128 (118–144)	< 0.05
P (mmol/L)	1.16 (1.04–1.29)	1.12 (1.03–1.23)	0.11
Cl (mmol/L)	105 (103–107)	104 (101–106)	< 0.05
Duration of T2D (years)	10 (4–20)	15 (11–21)	< 0.05
FIB (g/L)	2.97 (2.61–3.45)	3.07 (2.58–3.76)	0.15
PR (times/min)	79 (74–88)	84 (75–96)	< 0.05
PLT (10^9^/L)	202 (160–238)	190 (155–229)	0.22
Ca (mmol/L)	2.26 (2.18–2.34)	2.31 (2.23–2.39)	< 0.05
Na (mmol/L)	141 (139–142)	141 (139–143)	0.61
K (mmol/L)	3.9 (3.8–4.2)	4 (3.8–4.3)	< 0.05

*Note:* Continuous variables are presented as median (25th–75th percentile), with *p* value calculated using the Mann–Whitney *U* test. Categorical variables are expressed as percentages, with *p* value determined by a chi‐square test.

Abbreviations: 24‐h UTP, 24‐h urine total protein; 25(OH)D, 25‐hydroxyvitamin D; a1‐MG, a1‐microglobulin; BUN, blood urea nitrogen; Ca, calcium; Cl, chlorine; DPN, diabetic peripheral neuropathy; DPVD, diabetic peripheral vascular disease; DR, diabetic retinopathy; FIB, fibrinogen; HDL‐C, high‐density lipoprotein cholesterol; K, potassium; Na, sodium; P, phosphate; PLT, platelets; PR, pulse rate; RBP, retinol‐binding protein; RR, respiratory rate; SBP, systolic blood pressure; TBIL, total bilirubin; TC, total cholesterol.

### 3.3. Construction and Evaluation of the DKD Prediction Model

**Figure 5:**
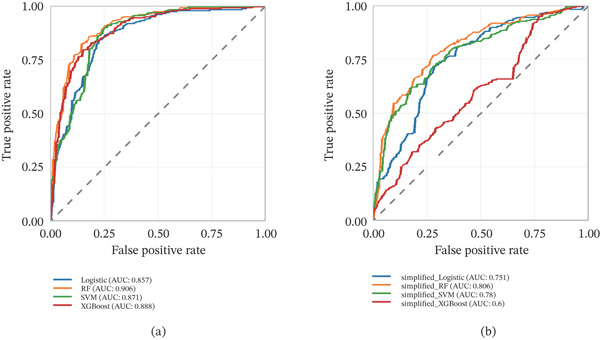
(a) ROC curves of the four models in the test set. (b) ROC curves of the four simplified models in the test set. Logistic, logistic regression; ROC, receiver operating characteristic; RF, random forest; SVM, support vector machine; XGBoost, extreme gradient boosting machine.

**Figure 6:**
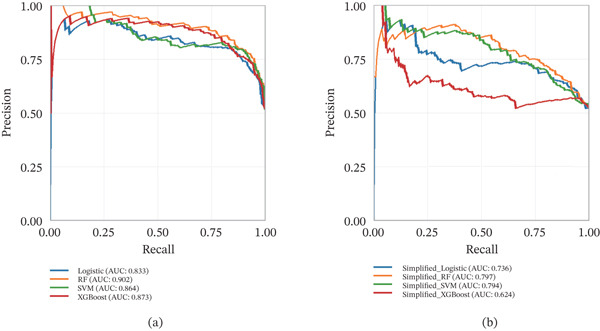
(a) PR curves of the four models in the test set. (b) PR curves of the four simplified models in the test set. Logistic, logistic regression; PR, precision‐recall; RF, random forest; SVM, support vector machine; XGBoost, extreme gradient boosting machine.

**Figure 7:**
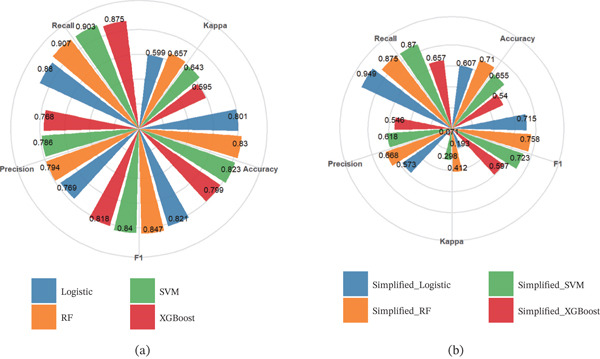
(a) Bar plots of each evaluation metric for the four models in the test set. (b) Bar plots of each evaluation metric for the four simplified models in the test set. Logistic, logistic regression; RF, random forest; SVM, support vector machine; XGBoost, extreme gradient boosting machine.

In this study, the hyperparameters confirmed through 10‐fold cross‐validation were used to construct the final models in the training set. The specific hyperparameter values are provided in Supporting Information (Table S1). Based on the test dataset, the four full classifiers of SVM, RF, XGBoost, and logistic regression used to classify DKD were evaluated, which showed that the full RF model (AUC‐ROC = 0.906, AUC‐PR = 0.902, accuracy = 0.830, F1 = 0.847, precision = 0.794, recall = 0.907, and kappa = 0.657) displayed the most excellent predictive performance (Figures 5, 6, and 7).

**Figure 8:**
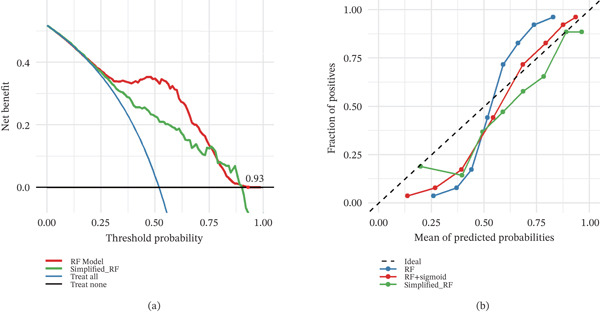
(a) Clinical utility of the RF model and the simplified RF model. The decision curve presents three scenarios: The solid black line represents intervening on no patients, the blue line represents intervening on all patients, and the red line and the green line depict the net benefit of using the RF model and the simplified RF model to guide interventions, respectively. (b) Calibration curves of the RF model and the simplified RF model. The blue and red lines represent the pre‐ and postcalibration performance of the RF model, respectively; the green line represents the performance of the simplified RF model, and the dashed line indicates full calibration. RF, random forest.

Four clinical simplified models were also evaluated on the test set. Among these, the simplified RF model achieved the highest AUC‐ROC of 0.806 (Figure 5b). However, its performance was significantly inferior to that of the full RF model (*p* < 0.001, DeLong’s test). The full RF model demonstrated a higher net benefit than the simplified model across threshold probabilities ranging from 0.25 to 0.75 (Figure 8a), indicating a clinically meaningful improvement in decision‐making. Furthermore, the calibration of the full RF model was superior to that of the simplified model (Brier score: 0.155 vs. 0.193; Figure 8b).

As shown in Figure 8a, the full RF model maintained a positive net benefit across threshold probabilities from 0% to 93%. Predicted probabilities on the test set were calibrated using the Platt scaling method, and the resulting calibration curve is presented in Figure 8b. After calibration, the calibration curve of the RF model progressively approached the diagonal line, indicating that the model’s predictive performance aligned closely with actual outcomes and thus possessed practical utility.

### 3.4. Explanation Analysis of the RF Model

**Figure 9:**
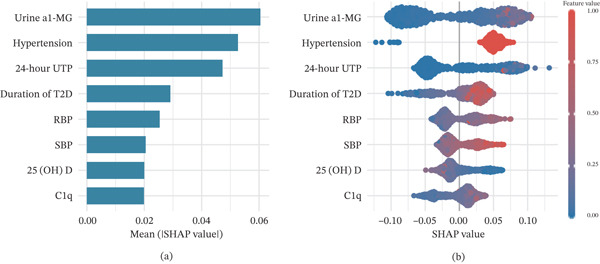
Explanation analysis of the RF model. (a) The importance of various features in the RF model. (b) RF model interpretability based on SHAP values. This plot shows the Shapley values (*x*‐axis) of each feature (*y*‐axis), which are ordered by importance. Feature values are indicated by color, ranging from low to high. SHAP, SHapley Additive exPlanations.

The contribution of individual features to the screening score was assessed using SHAP, a method grounded in cooperative game theory that quantifies feature importance on the test dataset. A positive Shapley value of a feature suggests an association with an elevated risk of DKD, whereas a negative value indicates a reduced risk. Figure 9 shows the importance of SHAP values and a global summary of the top importance features. Urine a1‐MG, hypertension, 24‐h UTP, duration of T2D, RBP, SBP, 25(OH)D, and C1q were identified as the Top 8 metrics affecting the identification of DKD from T2D, of which serum 25(OH)D was negatively correlated with DKD, and the rest were positively correlated.

**Figure 10:**
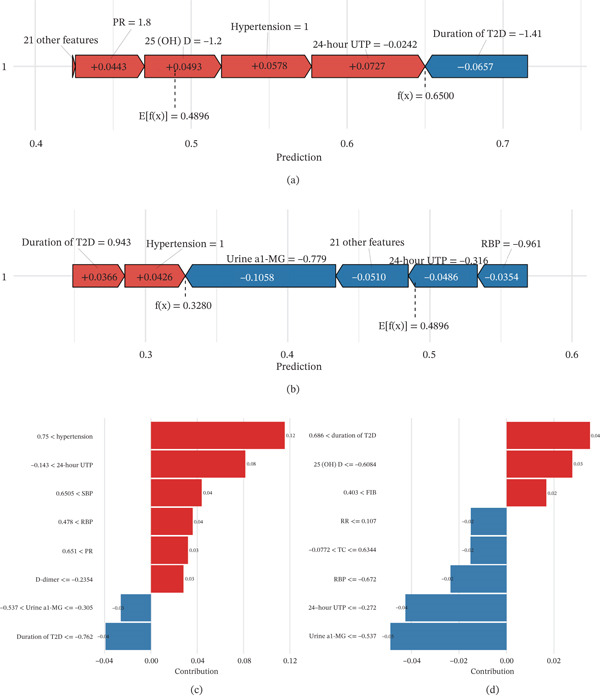
Two examples of local interpretability visualization using SHAP values and the LIME method. (a) A subject with a diabetes duration of 2 years. (b) A subject with a diabetes duration of 20 years. For these SHAP‐based plots, features that elevate the predicted score above the baseline (mean prediction) are shown in red, while those that lower the prediction relative to the baseline are shown in blue. (c) A subject with a diabetes duration of 2 years. (d) A subject with a diabetes duration of 20 years. For these LIME‐based plots, red features support the prediction of DKD, and blue features support the prediction of the opposite class. The feature weight indicates the magnitude of each feature’s contribution to the final prediction.

LIME provides local interpretability by fitting a sparse linear model to predictions on perturbed instances near the input, approximating the local decision boundary. This contrasts with SHAP, which relies on Shapley values. Both methods were applied to two illustrative test cases. Case 1, with short diabetes duration but multiple abnormal parameters (PR, 25(OH)D, hypertension, and 24‐h UTP), received a high predicted DKD risk (Figure 10a). Case 2, despite long‐standing diabetes, displayed largely normal values for these features and a correspondingly low risk (Figure 10b). LIME and SHAP attributions differed in detail but converged on consistent risk assessments (Figure 10c,d).

### 3.5. Assessment of Selection Bias and Robustness to Missing Data

To evaluate the potential for selection bias, baseline characteristics were contrasted between patients retained in the training cohort (*n* = 1046) and those excluded (*n* = 295). As detailed in Table S2, no statistically significant disparities emerged for gender, DKD, DPN, DPVD, DR, or hypertension (all *p* > 0.05), indicating negligible selection bias. Diabetes duration, however, exhibited a significant intergroup difference (*p* < 0.05). Comparable patterns were observed in the test cohort (Table S3). The robustness of the primary findings to missing data handling was further examined through a sensitivity analysis employing median imputation. For the full RF model, the AUC‐ROC following median imputation reached 0.919, relative to 0.906 in the complete‐case analysis. These outcomes affirm that the principal conclusions remain insensitive to the method of addressing missingness, and that imputation did not substantively influence the results.

## 4. Discussion

Due to the complex and insidious pathogenesis of DKD, early diagnosis and intervention remain challenging. Single clinical indicator often lacks the sensitivity and specificity required for accurate diagnosis. In this study, we constructed four ML models to identify DKD in T2D based on kinds of clinical and laboratorial indicators to provide assistance in the early diagnosis and prevention.

Our findings demonstrated that the RF model outperformed other models, achieving an AUC‐ROC of 0.906 in the test set. Calibration curve analysis and DCA further indicated that the model possessed excellent calibration and clinical utility, highlighting its strong potential as a practical tool to detect DKD. A key strength of our study is the inclusion of a clinically realistic benchmark. Our model showed excellent improvement over a simplified model using only six routine variables; this finding highlights that ML can add value beyond routine clinical variables. Moreover, model interpretation revealed that urine *α*1‐MG was the most effective predictor in identifying DKD. Other significant factors included hypertension, 24‐h UTP, duration of T2D, RBP, SBP, C1q, and 25(OH)D. However, urine *α*1‐MG, 24‐h UTP, C1q, and RBP are not routinely measured in many settings and may be costly or logistically challenging. In such cases, the simplified RF model (AUC‐ROC = 0.806) could serve as a practical substitute for the full model.

Glomerular injury has long been considered central to the pathogenesis of DKD. However, emerging evidence highlights the morphological and functional changes of renal tubular in DKD, which are not the aftermath of glomerular damage but rather the primary results of diverse pathophysiological influences [19]. *α*1‐MG is a low‐molecular‐weight glycoprotein that functions as a hydrophobic ligand‐binding protein [20]. The free form of *α*1‐MG is readily filtered through the glomeruli but is almost completely reabsorbed and catabolized by the renal tubule [21]. Therefore, elevated urinary excretion of *α*1‐MG serves as a marker of impaired proximal tubular function. A study has demonstrated that urinary *α*1‐MG is independently associated with eGFR, indicating its potential significant role in the pathogenesis of DKD [22]. Nevertheless, the role of urinary *α*1‐MG as a ML predictor has not been explored. Our study reveals its predictive value in the onset of DKD.

RBP is a carrier protein composed of carbohydrates and a polypeptide chain. It has a very short half‐life and binds to thyroid transporter proteins to form a polymeric complex in plasma. The activated form of RBP circulates freely in plasma and is filtered by the glomeruli. Most filtered RBP is reabsorbed and degraded by the proximal tubules for tissue utilization, with only minimal amounts being excreted in the urine [21]. Therefore, under physiological conditions, RBP levels in both serum and urine are exceedingly low. Our results support that serum RBP can act as a reliable predictor of DKD, which is in agreement with previous studies [23].

Activation of the complement system has been documented in patients with diabetic nephropathy. C1q deposits within the glomerular hila and arterioles were detected more frequently in DKD patients compared to non‐DKD individuals [24]. Moreover, the presence of C1q deposition was associated with significantly higher levels of urinary protein and lower eGFRs, indicating that complement deposition of C1q on renal histopathology is correlated with more severe renal impairment in DKD [25]. Although renal C1q deposition is well‐studied, the prognostic value of serum C1q in DKD remains to be elucidated. Our study demonstrates significantly elevated serum C1q concentrations in patients with DKD compared to diabetic individuals without renal involvement, indicating its promising potential as a predictive biomarker.

Vitamin D, a hormone integral to numerous physiological processes, has garnered increasing evidence supporting its renoprotective effects [26]. It modulates endothelial function, contributes to podocyte preservation, regulates the renin–angiotensin–aldosterone system, and exerts anti‐inflammatory actions [26]. Our results show that patients with DKD have lower serum Vitamin D levels than diabetics without nephropathy, which is consistent with previous studies [27, 28]. Supplementation with Vitamin D has been shown to benefit individuals with DKD by positively influencing key disease‐related markers, including reduction of proteinuria and creatinine levels [29]. Therefore, maintaining and monitoring serum Vitamin D within an optimal range may play a critical role in preventing DKD among patients with T2D.

In conclusion, this study developed and validated a risk prediction model for the diagnosis of DKD, which demonstrated favorable discriminatory ability in the test sets. This model offers a useful tool to facilitate early diagnosis and intervention, potentially helping to slow disease progression and mitigate its clinical impact. However, several limitations should be acknowledged. First, although the model exhibited robust performance, it remains subject to certain misclassification rates, particularly for predictions near intermediate risk thresholds. Second, as a case‐control study, the findings may be influenced by inherent selection bias, which could affect generalizability to the broader DKD population. The comparison of included versus excluded patients revealed a small difference, but a median imputation sensitivity analysis confirmed robustness, suggesting that selection bias is unlikely to drive our conclusions. Furthermore, a key limitation of our study is the lack of external validation. Although we used a temporal split to assess stability over time within our center, this remains an internal validation. The model was developed and tested on data from a single institution and a single regional population, which may not represent the diversity of patient demographics, clinical practices, or data acquisition systems in other hospitals or countries.

External validation in independent cohorts is essential to assess generalizability. We therefore plan to validate our model using data from at least two hospitals in different geographic regions. The existing model will be applied to both prospectively and retrospectively collected data from these sites, with evaluation of discrimination (AUC), calibration (calibration plot), and clinical utility (DCA). A minimum of 200 events and 200 nonevents will be required. Until such external validation is completed, the model should be considered exploratory and not ready for clinical deployment.

## 5. Conclusions

In summary, our study constructs an RF model with robust predictive performance to screen for DKD. Urine *α*1‐MG, hypertension, 24‐h UTP, duration of T2D, RBP, SBP, C1q, and 25(OH)D are revealed to be highly correlated with DKD risk. Our study provides a clue for the application of clinical and laboratory data to detect DKD in T2D.

## Author Contributions

Tongtong Qiu: conceptualization, formal analysis, investigation, methodology, software, visualization, and writing—original draft. Yi Bai: data curation, investigation, and writing—review and editing. Hai Zhao: methodology, software, and writing—review and editing. Mengyao Huang: data curation, resources, and writing—review and editing. Ping Zhang: investigation, validation, and writing—review and editing. Yu Zhang: investigation, validation, and writing—review and editing. Ruoyu Wang: investigation, validation, and writing—review and editing. Liqin An: conceptualization, funding acquisition, project administration, supervision, visualization, and writing—original draft.

## Funding

This work was financially supported by the National Youth Science Fund Project of the National Natural Science Foundation of China (Grant Number 82400441) and the Natural Science Basic Research Program of Shaanxi Province of China (Grant Number 2023‐JC‐QN‐0848).

## Disclosure

The funders had no role in the study design, data collection, data analysis, interpretation of results, writing of the manuscript, or the decision to submit the article for publication.

## Ethics Statement

Ethical approval was granted by the Ethics Committee of Shaanxi Provincial People’s Hospital (Approval No. SPPH‐LLBG‐34‐4.0‐2025R078).

## Conflicts of Interest

The authors declare no conflicts of interest.

## Supporting information


**Supporting Information** Additional supporting information can be found online in the Supporting Information section. Table S1. Optimized hyperparameters and corresponding AUC‐ROC values of four machine learning models (SVM, XGBoost, RF, and logistic) used in the study. The AUC‐ROC score served as the criterion for model selection, with the optimal model being the one that yielded the maximum value. Table S2. Comparison of baseline characteristics between included (*n* = 1046) and excluded (*n* = 295) patients in the training cohort. No statistically significant disparities emerged for gender, DKD, DPN, DPVD, DR, or hypertension (all *p* > 0.05). Diabetes duration, however, exhibited a significant intergroup difference (*p* < 0.05). Table S3. Comparison of baseline characteristics between included (*n* = 417) and excluded (*n* = 92) patients in the test cohort. No statistically significant disparities emerged for gender, DKD, DPN, DPVD, DR, or hypertension (all *p* > 0.05). Diabetes duration, however, exhibited a significant intergroup difference (*p* < 0.05).

## Data Availability

The data that support the findings of this study are available from the corresponding author upon reasonable request.
