# Supplementary material for: Identifying Diabetic Kidney Disease in Type 2 Diabetes Patients Using Explainable Machine Learning: A Case‐Control Study
Source: J Diabetes Res. 2026 Jun 7;2026:7196309. doi: 10.1155/jdr/7196309 (PMC13243769; doi:10.1155/jdr/7196309)
Supplement: Supplementary file 1 — Supporting Information Additional supporting information can be found online in the Supporting Information section. Table S1. Optimized hyperparameters and corresponding AUC‐ROC values of four machine learning models (SVM, XGBoost, RF, and logistic) used in the study. The AUC‐ROC score served as the criterion for model selection, with the optimal model being the one that yielded the maximum value. Table S2. Comparison of baseline characteristics between included (n = 1046) and excluded (n = 295) patients in the training cohort. No statistically significant disparities emerged for gender, DKD, DPN, DPVD, DR, or hypertension (all p > 0.05). Diabetes duration, however, exhibited a significant intergroup difference (p < 0.05). Table S3. Comparison of baseline characteristics between included (n = 417) and excluded (n = 92) patients in the test cohort. No statistically significant disparities emerged for gender, DKD, DPN, DPVD, DR, or hypertension (all p > 0.05). Diabetes duration, however, exhibited a significant intergroup difference (p < 0.05). [file JDR-2026-7196309-s001.docx]

**Supplementary**

Table S1 The optimized hyperparameters of four machine learning models

| Classifiers | Adjusted hyperparameters | AUC-ROC |
| --- | --- | --- |
| SVM | sigma = 0.01 and C = 1. | 0.829 |
| XGBoost | nrounds=50, max_depth=6, eta=0.1, gamma=1, colsample_bytree = 0.7, min_child_weight = 3, subsample = 0.7 | 0.843 |
| RF | mtry = 2, ntree = 500 | 0.848 |
| Logistic |  | 0.842 |

The hyperparameter tuning process employed 10-fold cross-validation and a grid search. The AUC-ROC score served as the criterion for model selection, with the optimal model being the one that yielded the maximum value. Abbreviations: RF: random forest; SVM: support vector machine; XGB: extreme gradient boosting. AUC-ROC: area under the receiver operating characteristic curve.

Table S2 Comparison of included and excluded patients in the training cohort

|  | Inclusion (1046) | Exclusion (295) | *p-*value |
| --- | --- | --- | --- |
| Male gender, *n (%)* | 680 (65.0%) | 208 (70.5%) | 0.08 |
| Duration of T2D (years)  DKD, *n (%)*  DPN, *n (%)* | 11.79 ±7.58  513 (49.0%)  545 (52.1%) | 10.95 ±5.86  145 (49.2%)  142 (48.1%) | <0.05  0.97  0.22 |
| DPVD, *n (%)* | 568 (54.3%) | 157 (53.2%) | 0.74 |
| DR, *n (%)* | 168 (16.1%) | 40 (13.6%) | 0.29 |
| Hypertension, *n (%)* | 674 (64.4%) | 180 (61.0%) | 0.28 |

Continuous variables are presented as mean ± standard deviation, with p-value calculated using the Student's t-test. Categorical variables are expressed as percentages, with p-value determined by a chi-square test. Abbreviations: DPN: diabetic peripheral neuropathy; DPVD: diabetic peripheral vascular disease; DR: diabetic retinopathy; DKD: Diabetic kidney disease.

Table S3 Comparison of included and excluded patients in the test cohort

|  | Inclusion (417) | Exclusion (92) | *p-*value |
| --- | --- | --- | --- |
| Male gender, *n (%)* | 266 (63.8%) | 61 (66.3%) | 0.65 |
| Duration of T2D (years)  DKD, *n (%)*  DPN, *n (%)* | 14.08 ±8.50  216 (51.8%)  147 (35.3%) | 11.06±5.88  43 (46.7%)  27 (29.3%) | <0.05  0.38  0.28 |
| DPVD, *n (%)* | 119 (28.5%) | 22 (23.9%) | 0.37 |
| DR, *n (%)* | 77 (18.5%) | 12 (13.0%) | 0.22 |
| Hypertension, *n (%)* | 407 (97.6%) | 89 (96.7%) | 0.64 |

Continuous variables are presented as mean ± standard deviation, with p-value calculated using the Student's t-test. Categorical variables are expressed as percentages, with p-value determined by a chi-square test. Abbreviations: DPN: diabetic peripheral neuropathy; DPVD: diabetic peripheral vascular disease; DR: diabetic retinopathy; DKD: Diabetic kidney disease.
